# Physical Gels of Atactic Poly(N-isopropylacrylamide) in Water: Rheological Properties and As-Derived Spinodal Temperature

**DOI:** 10.3390/gels9040288

**Published:** 2023-04-01

**Authors:** Ya-Chen Chuang, Yu Wang, Chi Wang

**Affiliations:** Department of Chemical Engineering, National Cheng Kung University, Tainan 70101, Taiwan

**Keywords:** physical gelation, phase separation, phase diagram, spinodal temperature, rheological properties

## Abstract

Aqueous solutions of atactic poly(N-isopropylacrylamide) (a-PNIPAM) undergo complex phase transitions at 20–33 °C. In this temperature range, the a-PNIPAM solution exhibits a phase behavior of lower critical solution temperature at the binodal temperature (*T*_b_) and physical gel formation at the gel temperature (*T*_gel_). On slow heating of the one-phase solution containing linear a-PNIPAM chains, branched chains are gradually developed to proceed with the physical gelation before phase separation considering that *T*_gel_ < *T*_b_. Thus, the phase separation temperature determined from the conventional approaches, either by turbidity to derive the *T*_b_ or by scattering to derive the spindal temperature (*T*_s_) from the Ornstein–Zernike analysis, is strictly the transition temperature associated with the a-PNIPAM hydrogel (or highly branched chains newly developed at elevated temperatures), rather than the initial a-PNIPAM solution prepared. Herein, the spinodal temperatures of a-PNIPAM hydrogels (*T*_s,gel_) of various concentrations were determined from rheological measurements at a heating rate of 0.2 °C/min. Analyses of the temperature dependence of loss modulus *G″* and storage modulus *G′* give rise to the *T*_s,gel_, based on the Fredrickson–Larson–Ajji–Choplin mean field theory. In addition, the specific temperature (*T*_1_) above which the one-phase solution starts to dramatically form the aggregated structure (e.g., branched chains) was also derived from the onset temperature of *G′* increase; this is because as solution temperature approaches the spinodal point, the concentration fluctuations become significant, which is manifested with the elastic response to enhance *G′* at *T* > *T*_1_. Depending on the solution concentration, the measured *T*_s,gel_ is approximately 5–10 °C higher than the derived *T*_1_. On the other hand, *T*_s,gel_ is independent of solution concentration to be constant at 32.8 °C. A phase diagram of the a-PNIPAM/H_2_O mixture is thoroughly constructed together with the previous data of *T*_gel_ and *T*_b_.

## 1. Introduction

Poly(N-isopropylacrylamide) (PNIPAM) is a widely used thermoresponsive polymer that has gained increasing attention in the past five decades [1,2,3,4,5,6,7,8]. In the aqueous solutions, PNIPAM chains are well-hydrated and swollen at temperatures below their lower critical solution temperature (LCST), which is approximately 31 °C. The good solubility of PNIPAM in water is primarily because of the cooperative hydration of the bound water molecules to form the “hydrogen bond bridge” around isopropyl groups to compensate for the entropy loss. When heated above its LCST, PNIPAM chains lose the bound water molecules to undergo a hydrophobic collapse, thereby forming a compact chain conformation; at this stage, the free energy change upon mixing of PNIPAM and water becomes positive, thereby leading to phase separation. Hence, it is concluded that hydrophobic hydration/dehydration plays a key role in the LCST behavior of PNIPAM/water solution. It is essential to understand the interplay of bound water and free water molecules around the PNIPAM chains to further grasp the LCST behavior of PNIPAM. To this end, Ono and Shikata [9] have revealed that approximately 11 bound water molecules are involved in forming the water bridge to enclose one repeating unit. Moreover, the number of bound water molecules per repeating unit remains intact until ~30 °C, above which dehydration occurs. In contrast with other polymer solutions, the LCST is independent of the average molecular weight of PNIPAM used. Okada and Tanaka [10] have derived the flat LCST curve of PNIPAM/water based on the pearl-necklace chain model together with the cooperative hydration concept, which was consistent with the experimental results. However, all the previous LCST measurements did not consider the physical gelation of the PNIPAM/water solution, which is likely to occur before the solution becomes phase-separated.

On the one hand, some preliminary evidence for the PNIPAM aqueous solution to proceed with the physical gelation has been reported in the work by Zeng et al. [11] using a rheological approach. On the other hand, Nakao et al. [12] also reported the physical gelation of isotactic-rich PNIPAM (i-PNIPAM) using a simple ball-drop method. A recent work by Kawaguchi et al. [13] demonstrated that the conformation of PNIPAM chains varied by the increasing temperature at *T* lower than the cloud point (*T*_cp_), thereby suggesting that branched chains are gradually developed before solution phase separation. The occurrence of physical gelation of the atactic PNIPAM (a-PNIPAM) with a molecular weight of 6.58 × 10^5^ g/mol is unambiguously disclosed by the rheological approach based on the Winter-Chambon criterion of the frequency-independency of loss tangent [14]. Several merits deserved to be emphasized as follows: (1) the derived gel point (GP, i.e., the *T*_gel_) decreases with increasing a-PNIPAM concentration, (2) *T*_gel_ is lower than the binodal temperature (*T*_b_) and the gap between them increases with increasing a-PNIPAM concentration, (3) the critical relaxation exponent (*n*) at GP is constant at 0.758, regardless of the a-PNIPAM concentration, and (4) the developed gel is extremely soft with a low modulus of approximately 10 Pa, thereby suggesting that many primitive a-PNIPAM chains are involved in between two neighboring physical junctions.

Given that *T*_gel_ < *T*_b_, the two transition processes (i.e., the physical gelation and phase separation) are expected to sequentially occur when a one-phase solution is subjected to continuous heating to high temperatures; the one-phase a-PNIPAM/H_2_O solution at low temperatures will initially form a macroscopic a-PNIPAM gel at *T* > *T*_gel_, and the transparent a-PNIPAM gel eventually becomes phase-separated at *T* > *T*_b_. Thus, the conventional methods used to determine the phase separation temperature (either *T*_b_ or spinodal temperature, *T*_s_) by heating from the one-phase solution domain are to derive the *T*_b_ and *T*_s_ of the a-PNIPAM hydrogel, not the original one-phase solution prepared.

Various advanced techniques have been applied to determine the phase-separation temperature of the PNIPAM/water solution. The mechanism of phase separation was extensively investigated in the past five decades [1,2,3,4,5,6,7,8]. Turbidity measurements at different heating rates are performed to reveal the heating-rate dependence of *T*_cp_. Afterward, *T*_b_ is preferentially obtained from the extrapolated *T*_cp_ at a zero-heating rate to construct the coexistence curve [14,15]. On the contrary, the *T*_s_ of the polymer solutions is often determined from the small-angle X-ray scattering (SAXS) [16] and neutron scattering (SANS) [17,18]. The scattered intensity profiles of PNIPAM/H_2_O are obtained in the one-phase domains at different *T* (<*T*_b_) by stepwise heating, followed by the rigorous scattered intensity analysis primarily based on the Ornstein-Zernike scattering function. Alternatively, the *T*_s_ of PNIPAM/H_2_O solutions could also be obtained by time-resolved light scattering (TRLS) via a temperature-jump approach from the one-phase domain at *T* < *T*_b_ to the two-phase domain at *T* > *T*_b_ [19]; the obtained TRLS intensity profiles are further analyzed based on the Cahn theory. Despite extensive studies in the past decades, the determination of *T*_b_ (and *T*_s_) of PNIPAM/water remains very elusive considering that the effect of physical gelation caused by chain associations is often neglected. A collective review focusing on the phase separation and the related phase diagram of PNIPAM/H_2_O may provide an in-depth discussion for interested readers [3].

In addition to the scattering approach, rheometry is a powerful tool to trace the subtle structure variation of polymer blends undergoing the liquid-liquid phase separation [20]. Recently, progress in elucidating phase separation of polymer blends (and polymer solutions) to derive *T*_s_ was achieved by monitoring viscoelastic properties during the phase transition [21,22,23,24]. In principle, *T*_s_ can be deduced from the evolution of dynamic storage and loss moduli obtained from a dynamic temperature sweep test, and the derived *T*_s_ by rheometry is consistent with the conventional scattering approach. Previously, we have performed the turbidity and rheological measurements of a-PNIPAM aqueous solutions to obtain the transition temperatures of *T*_b_ and *T*_gel_, respectively [14,15]. In this work, a dynamic temperature sweep test was performed on the a-PNIPAM/H_2_O with various concentrations to deduce the corresponding *T*_s_ and *T*_1_; the latter is the transition temperature above which the one-phase solution reaches the pre-gel solution regime with enhanced elasticity. For the first time, a precise phase diagram of a-PNIPAM aqueous solution is constructed based on these transition temperatures.

## 2. Results and Discussion

**Figure 1:**
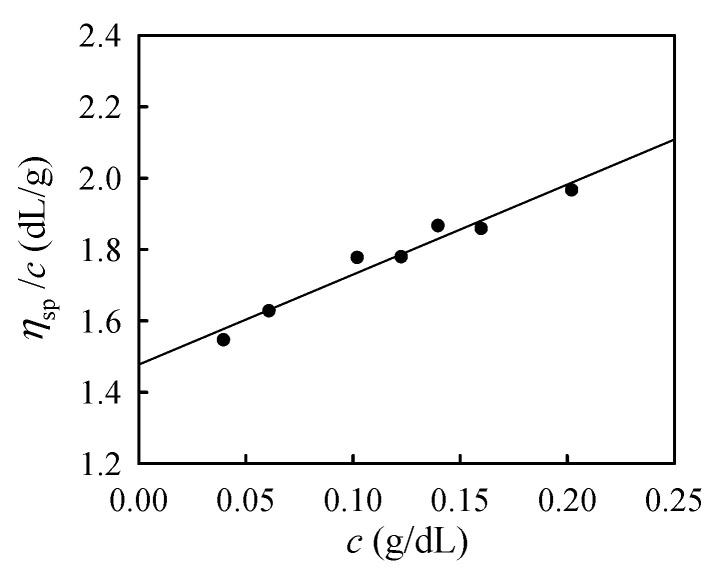
Plots of *η*_sp_/c versus *c* at 20 °C to determine the intrinsic viscosity of a-PNIPAM/H_2_O solution from the intercept at *c* = 0.

The polymer concentration should be higher than the overlap concentration ϕ* to facilitate chain associations in the overlapping region to develop a physical gelation between a-PNIPAM chains. ϕ* can be estimated by the inverse of the intrinsic viscosity, that is, ≈[*η*]^−1^. Figure 1 shows the plot of *η*_sp_/*c* versus *c* at 20 °C, where *η*_sp_ and *c* are the specific viscosity and polymer concentration, respectively. Based on the Huggins equation *η*_sp_/*c =* [*η*] *+ k’*[*η*]^2^ *c*, the extrapolated intercept at *c* = 0 gives rise to the [*η*] of 148.8 mL/g. Thus, the ϕ* of the present a-PNIPAM in de-ionized water is approximately 0.6 wt%. The entanglement concentration *ϕ*_e_ has been determined at 8 wt% [14,15]. In this work, a-PNIPAM solutions with various concentrations (5–12 wt%) were used to investigate their rheological properties, from which the corresponding *T*_s_ was determined.

### 2.1. Rheological Curves Obtained from Temperature Sweep Test

**Figure 2:**
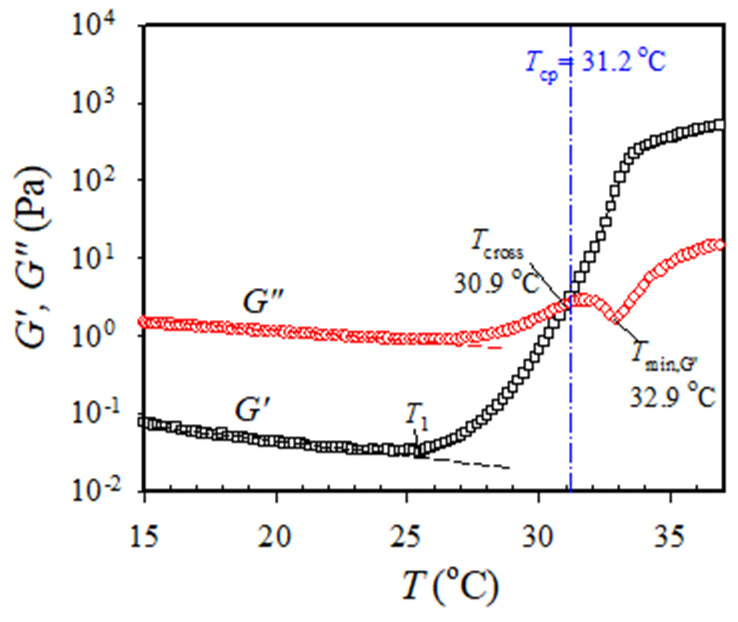
Dynamic storage modulus *G′* and loss modulus *G″* of the 7 wt% a-PNIPAM aqueous solution during the temperature sweep test. The onset temperature of *G′* upturn is denoted by *T*_1_. Both the crossover temperature (*T*_cross_) and the temperature at the *G″* local minimum (*T*_min,G”_) are displayed. The dashed lines are for the eye-guide to illuminate the onset of upturn in *G′* and *G″* vs. *T*. The heating rate is 0.2 °C/min and the applied frequency is 0.5 rad/s. *T*_cp_ determined from the turbidity measurement at a heating rate of 0.2 °C/min is shown by the dash–dot line.

Figure 2 shows the typical *G′* and *G″* variation obtained from the temperature sweep test for the 7 wt% a-PNIPAM solution subjected to an applied frequency of 5 rad/s at a heating rate of 0.2 °C/min. The linear viscoelastic response was ascertained under a strain amplitude of 10% at the temperature range of 10–40 °C. Upon heating from 10 °C, *G′* and *G″* gradually decreased with increasing temperature until 25–26 °C, at which an upturn is observed. The upturn of *G′* happens slightly earlier than the *G″*; the corresponding temperature is designated by *T*_1_. At the temperature range of 10–25 °C, *G″* is one order magnitude higher than *G′*, thereby inferring that viscous nature is dominant over elastic nature. There is an expected continuous decrease of *G′* and *G″* with *T* which is attributed to the enhanced chain mobility at high temperatures. The upturn of *G′* and *G″* curves with *T* indicates that the statistical mechanical characteristics of the solution in the single phase have been altered as follows. At *T* > *T*_1_, the solution starts to have such enhanced concentration fluctuations that must fulfill the criterion of their characteristic relaxation times being larger than the time scale of the observation specified by 1/*ω*. Such thermal concentration fluctuations are developed at temperatures much below the cloud point of 31.2 °C determined from the turbidity at 0.2 °C/min heating as shown by the vertical dash–dot line.

The onset temperature at which *G′* upturns with *T* indicates the onset of the enhanced concentration fluctuations to develop the branched chains by chain association [14], thereby, implying that the one-phase solution at *T* < *T*_1_ eventually reaches the pre-gel regime at the temperature of *T*_1_. Remarkably, a continuous and significant increase in *G′* with a three-order magnitude enhancement at the temperature range of 25–33 °C was observed. The increase in *G′* becomes mild above 33 °C. In contrast, the *G″* curve at the temperature range of 25–33 °C exhibits distinct temperature dependence, whereby an evident *G″* minimum is seen at 32.9 °C (denoted by *T*_min,G”_), immediately followed by a second *G″* uprise. More importantly, a crossover of the *G′* versus *T* curve and *G″* versus *T* curve is detected at 30.9 °C, above which *G′* becomes dominant over *G″*. The crossover temperature is denoted by *T*_cross_, thereby inferring the occurrence of macroscopic physical gelation. However, it should be reminded that *T*_cross_ is not the gel point (GP at *T*_gel_) given that the critical relaxation exponent (*n*) for the present a-PNIPAM/H_2_O system is 0.758, regardless of the a-PNIPAM concentration [14]. It is reminded that *T*_cross_ can be strictly taken as *T*_gel_ only under the condition of a stoichiometrically balanced end-linking network with *n* = 0.5 [25].

**Figure 3:**
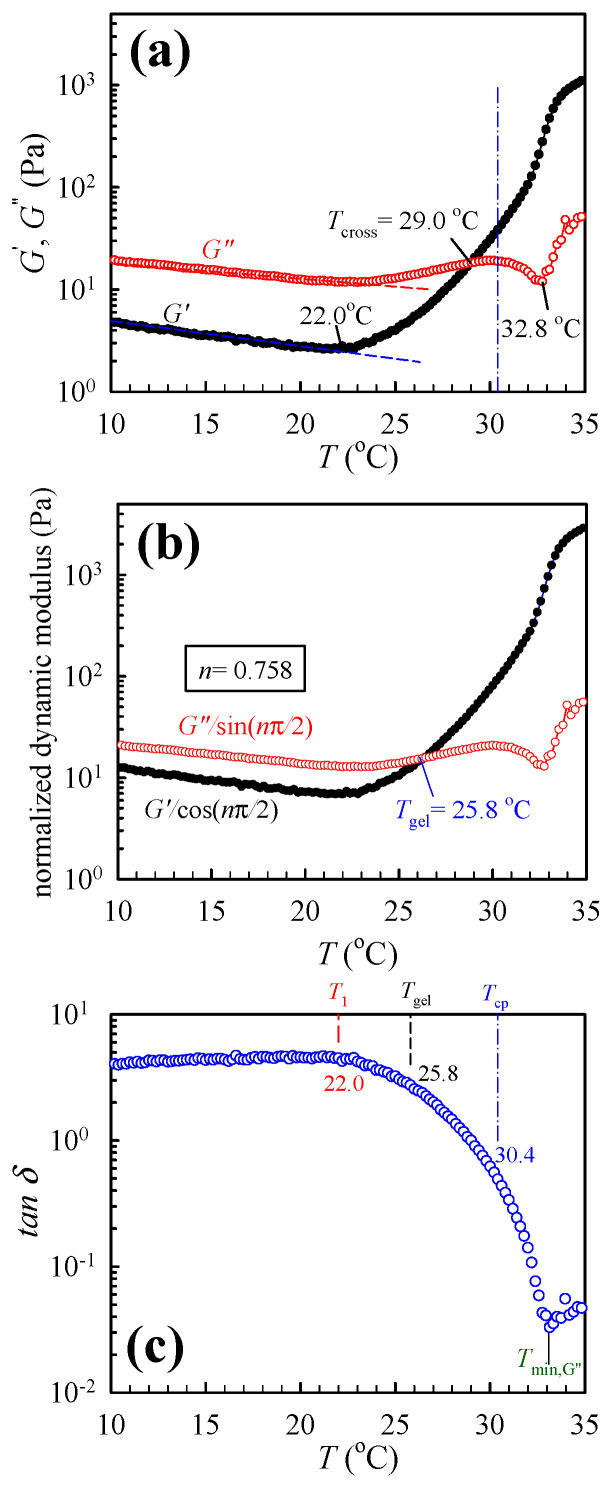
(**a**) Dynamic storage modulus *G′* and loss modulus *G″* of the 12 wt% a-PNIPAM aqueous solution during temperature sweep test, (**b**) the normalized curves of *G′* and *G″* with respect to a critical relaxation exponent *n* of 0.758, and (**c**) the loss tangent (=*G″/G′*). Both the *T*_cross_ and *T*_min,G”_ are displayed. The dashed lines are for the eye-guide to illuminate the onset of upturn in *G′* and *G″* vs. *T*. *T*_cp_ determined from the turbidity measurement is shown by the dash-dot line.

Similar trends are observed for the 12 wt% solutions (Figure 3a), whereby both the *T*_1_ and *T*_cross_ are lowered to 22.0 °C and 29.0 °C, respectively; however, the *T*_min,G”_ remains relatively unchanged at 32.8 °C. In addition, the *G′-G″* crossover temperature is 1.4 °C lower than the cloud point which is 30.4 °C. The magnitude of *tan δ* significantly decreases from 4.0 in the one-phase solution at *T* < *T*_1_ to 0.03 as the physical gel becomes phase-separated at *T* > *T*_cp,_ as shown in Figure 3c. A distinct minimum of *tan δ* could also be observed at *T*_min,G”_. For the crosslinking network with *n* ≠ 0.5, a convenient approach proposed by Winter [26] is applied to extract the *T*_gel_ by plotting the normalized dynamic moduli with a known value of *n*, that is, *G″*/sin(*n*π/2) and *G′*/cos(*n*π/2) as a function of *T*, from which the crossover temperature is the *T*_gel_. Such a plot is shown in Figure 3b, and the derived *T*_gel_ is 25.8 °C, which is consistent with the value of 25.0 °C obtained rigorously from the frequency-independence of *tan δ* analysis [14,15]. Thus, the *T*_cross_ obtained from the temperature sweep test is higher than the *T*_gel_ for the present a-PNIPAM/H_2_O solution, thereby suggesting that macroscopic gel has been already formed at *T* = *T*_cross_ before reaching the *T*_cp_.

The values of *G′* at *T*_cross_ are 2 and 18 Pa for the 7 and 12 wt% solutions, respectively. These values are approximately two orders of magnitude lower than the modulus of conventional hydrogels of ~1 kPa. Thus, a very soft a-PNIPAM gel is developed because of its extremely low “cross-linking” density, being 2.9 × 10^−3^ and 4.9 × 10^−2^ mol/m^3^ for the 7 and 12 wt% solutions, respectively [14]. The apparent molecular weight between two physical junctions is ca. 3 × 10^6^ − 2.5 × 10^8^ g/mol, thereby suggesting that at least 4–37 primitive a-PNIPAM chains are intermolecularly associated with one another to develop the single strand of the gel network. The shapes of a-PNIPAM gels may effectively change freely because of the low cross-linking density [25].

Each strand of the soft gel may be built up by dimers and trimers of single chains as its primary building blocks. The building blocks are further linked together into a long strand. Hence, the strand is composed of various single chains and is expected to be flexible. The dimers and trimers are composed of four and seven sub-chains in the case when the end-to-end associations of the single chains are less probable. The strands, the sub-chains in the strands, dimers, and trimers (or multimers in general) coexisting free from the strands, and single unassociated chains (unimers) may all have Gaussian-chain characteristics and indistinguishably contribute to the thermal concentration fluctuations. These unique features may indicate that upon heating, a-PNIPAM/water solutions undergo profound concentration fluctuations above *T*_1_. The lifetime of the fluctuations must be larger than the time scale of the *G′* measurements, that is, 1/*ω*~0.2 s. The enhanced thermal concentration fluctuations further trigger the physical gelation process to develop the soft gel before phase separation at *T* > *T*_cp_.

The upturn of *G′* is a general phenomenon that also occurs for polymer mixtures: the upturn may be attributed to (1) the thermal concentration fluctuations for the mixtures when the temperature approaches the spinodal point of the mixtures [20], and (2) the existence of domain interface because of the phase separation via nucleation/growth for the off-critical mixtures when crossing the binodal point [22]. Han et al. [22] considered the obtained *T*_1_ as the “apparent” binodal temperature and reported that *T*_1_ is elevated at a higher heating rate for the off-critical blends based on the extensive studies of the binary blends of polybutadiene/polyisoprene (PB/PI). In their case, the thermal concentration fluctuations become large only very near but below the binodal point in the single-phase mixture or above the binodal point wherein the phase separation starts to occur. In addition, the physical origin of *T*_1_ observed by them in their study on the PB/PI blends is quite different from the present a-PNIPAM/water solutions which form the macroscopic gel at a temperature lower than the cloud point as seen in Figure 2 and Figure 3.

**Figure 4:**
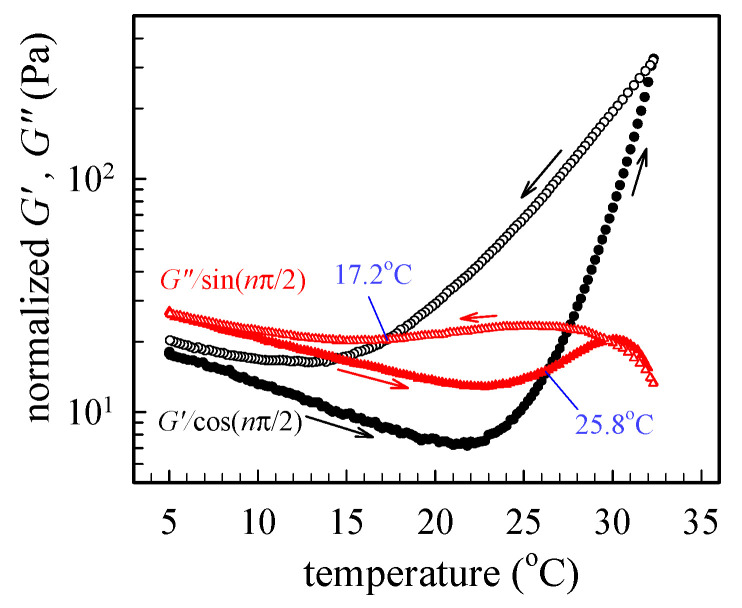
Hysteresis curves of normalized *G′* and *G″* of the 12 wt% solution upon 0.2 °C/min heating to 32 °C, followed by a subsequent 0.2 °C/min cooling. The filled and open symbols are data obtained during heating and cooling, respectively. The critical relaxation exponent (*n*) is 0.758.

The formation of physical gel is unambiguously confirmed by the large hysteresis loop, as shown in Figure 4, where the temperature dependence of *G′* for the 12 wt% solution subjected to 0.2 °C/min heating to 32 °C, immediately followed by 0.2 °C/min cooling. Both the heating and cooling curves fail to coincide, and a hysteresis loop is observed, thereby indicating that the rheological behavior in the cooling process is quite different from that in the heating process. The hysteresis loop observed is primarily caused by the imbalance of rate between the interchain association and dissociation of the hydrophobic N-isopropyl groups at different temperatures. These interchain associations eventually form the 3D gel junctions when the solution temperature is higher than *T*_gel_ (~25.8 °C). The gel junctions of the a-PNIPAM hydrogel primarily originated from the hydrophobic association between N-isopropyl groups [3,9]. “Hydrogen-bond breaking” between the water and amide group may further enhance the gel formation and its strength at elevated temperatures where cooperative dehydration occurs [9,10]. The pearl-necklace conformation of PNIPAM chains driven by the cooperative hydration implies that not all monomer units are hydrated along the backbone chain [10]. Moreover, the strength of hydrophobic hydration (i.e., the hydration of apolar entities of N-isopropyl) depends on the configuration or tacticity of the amphiphilic PNIPAM chains [12].

The magnitude of *G′* at 5 °C right after cooling is higher than that at 5 °C before heating, as seen in Figure 4. Thus, a long holding time at 5 °C is required to homogenize the cooled solution via interchain dissociation. The long relaxation time is because of the slow diffusion of water into the hydrophobic clusters (or gel junctions). Similar hysteresis effects have been observed on the melting enthalpy of PNIPAM aqueous solution measured by DSC heating/cooling trace [27,28]. In addition, the recovery time is much longer than the chemical gels of PNIPAM [28]. The difference in the hysteresis behavior between the chemical gels and physical gels of PNIPAM is likely related to the interchain associations and dissociations of the 3D network. The interchain associations in the chemical gels occur between the network strands. Thus, the associations involve a large cost of the elastic free energy of the network chains, which causes the associations less probable. On the contrary, in the physical gels, the interchain associations occur not only between the network strands but also between the network strands and the unimers or multimers, which are free from the strands. This makes the associations in the physical gels more extensive and intensive. Notably, a 3D transparent gel can be obtained until *T*_cp_ at temperatures higher than *T*_gel_, above which a turbid gel is expected.

The temperature dependence of *G*′ and *G″* curves (Figure 2 and Figure 3) may be altered if the solutions are subjected to different heating rates and applied frequencies. Thus, the effects of heating rate and applied frequency should be thoroughly investigated to extract meaningful material functions of *G*′ and *G″* for further analyses. In general, the heating rate for the system must be as low as possible and not too far from thermal equilibrium. Moreover, a low frequency is desirable to trace the evolution of large-scale structures with a long relaxation time.

### 2.2. Effects of Heating Rate on the Rheological Curves

**Figure 5:**
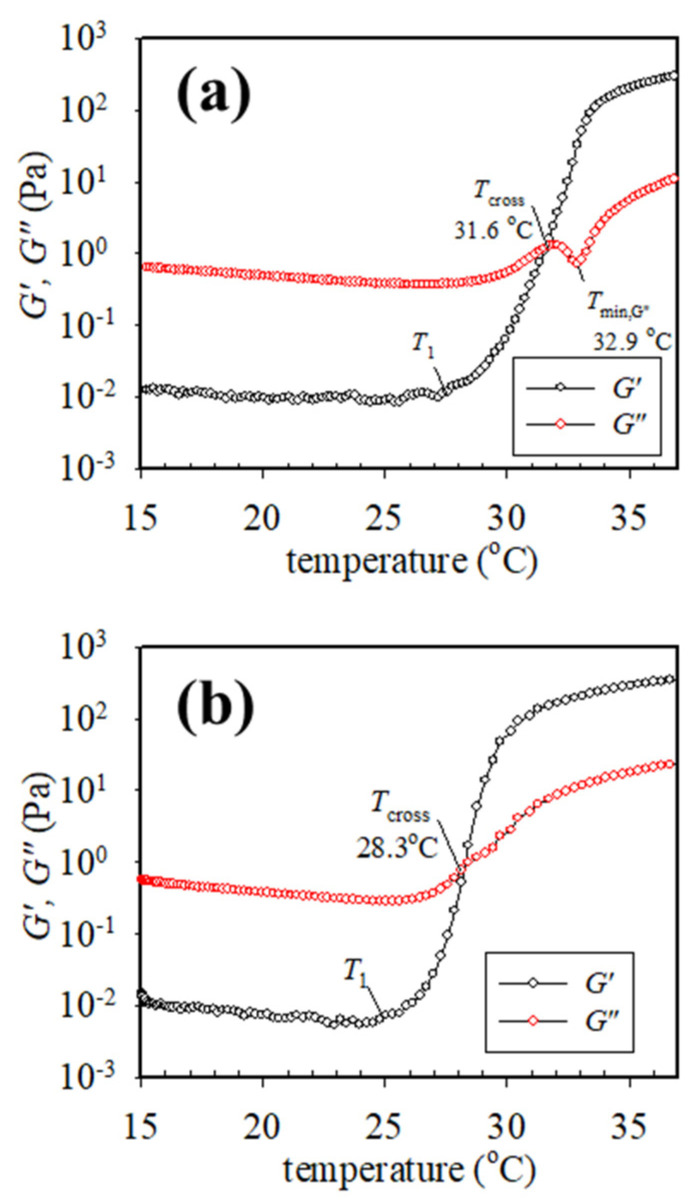
Temperature dependence of *G*′ and *G″* curves of the 5.5 wt% a-PNIPAM solution during a temperature sweep test at a heating rate of (**a**) 0.2 °C/min, and (**b**) 2.0 °C/min.

The 5.5 wt% solution was studied to reveal the heating rates on the obtained *G*′ and *G″* curves; the corresponding curves at a heating rate of 0.2 °C/min and 2.0 °C/min are illustrated in Figure 5a,b, with the same frequency of 5 rad/s, respectively. The most evident difference is the measured *G″* curves with the disappearance of the *T*_min,G”_ at a heating rate of 2.0 °C/min. Moreover, both *T*_cross_ and *T*_1_ decrease with increasing heating rates. These results indicate that heating rate plays an important effect on obtained rheological properties and selection of an appropriate heating rate is crucial.

**Figure 6:**
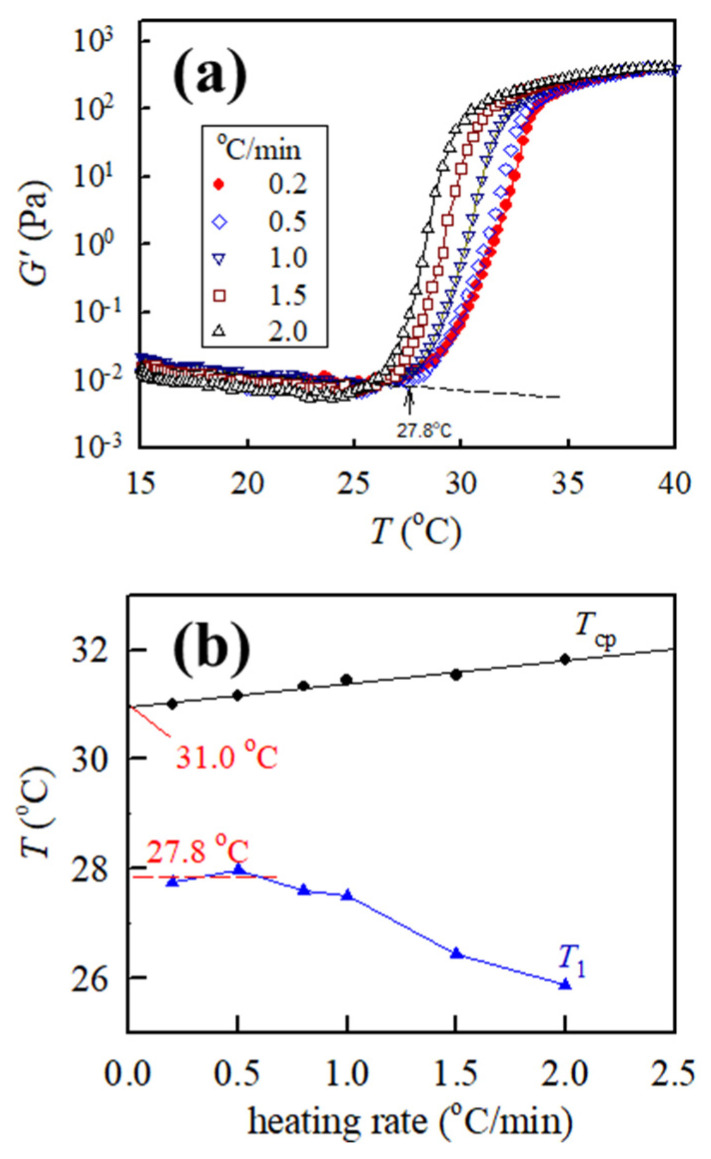
(**a**) Effects of heating rates on the dynamic storage modulus *G′* of the 5.5 wt% a-PNIPAM aqueous solutions at an applied frequency of 5 rad/s, and (**b**) heating rate effects on the obtained transition temperatures (*T*_1_ and *T*_cp_) of the 5.5 wt% solution.

Figure 6a shows the curves of *G*′ versus *T* for the 5.5 wt% a-PNIPAM solution obtained from different heating rates with the same frequency of 5 rad/s and strain amplitude of 10%. The 0.5 °C/min heating curve is marginally shifted to the low-temperature side, and the low-temperature shift becomes more pronounced at a higher heating rate (1.0 °C/min, 1.5 °C/min, and 2.0 °C/min) as compared with the heating curve at 0.2 °C/min. However, this trend is contrary to the turbidity measurement [14,15], thereby exhibiting that transmittance curves shift to the high-temperature side with an increased heating rate. Meanwhile, Figure 6b shows the plots of *T*_1_ and *T*_cp_ versus heating rate. The extrapolated value of *T*_cp_ at 0 heating rate is 31.0 °C, which is slightly lower than that determined from 0.2 °C/min heating (~31.4 °C). By contrast, *T*_1_ remains unchanged at ~27.8 °C at low heating rates of 0.2 °C/min and 0.5 °C/min but is gradually decreased with a further increase in heating rate. The drop of *T*_1_ at a heating rate higher than 0.5 °C/min is an artifact caused by the temperature measurement in the cup-and-bob fixture used in this study.

The measured *T*_cp_ is relevant to the phase separation via nucleation/growth, which requires a certain induction time to proceed. Hence, the measured *T*_cp_ depends upon the heating rate, whereby a higher heating rate delays the incipient indication in the nucleation/growth process up to a higher temperature. On the contrary, the determined *T*_1_ is likely relevant to the onset of remarkable concentration fluctuations when the solution approaches the spinodal point [20]. This criterion is particularly fulfilled for the critical solution, thereby exhibiting the so-called “critical slowing down” and “critical divergence” close to the critical point. The critical composition (*ϕ*_c_) for the a-PNIPAM/H_2_O solution is ~16 wt% [3]. Based on this conclusion, solutions with concentrations of 5–12 wt% studied in this work are off-critical. Even in this case, the contribution of thermal concentration fluctuations to enhance *G’* and *G”* may still play an important role in general in the single-phase solutions below *T*_cp_ (or *T*_gel_). Consequently, *T*_1_ should be independent of the heating rate applied. Indeed, we found that *T*_1_ remains relatively unchanged with the heating rate when it is lower than 0.5 °C/min. It seems that a heating rate of 0.2 °C/min used in this study is sufficient to extract the transition temperatures of *T*_1_ and *T*_cp_. A complex structure evolution is involved in the a-PNIPAM aqueous solutions in the temperature range from *T*_1_ to *T*_cp_.

### 2.3. Effects of Applied Frequency on the Rheological Curves

**Figure 7:**
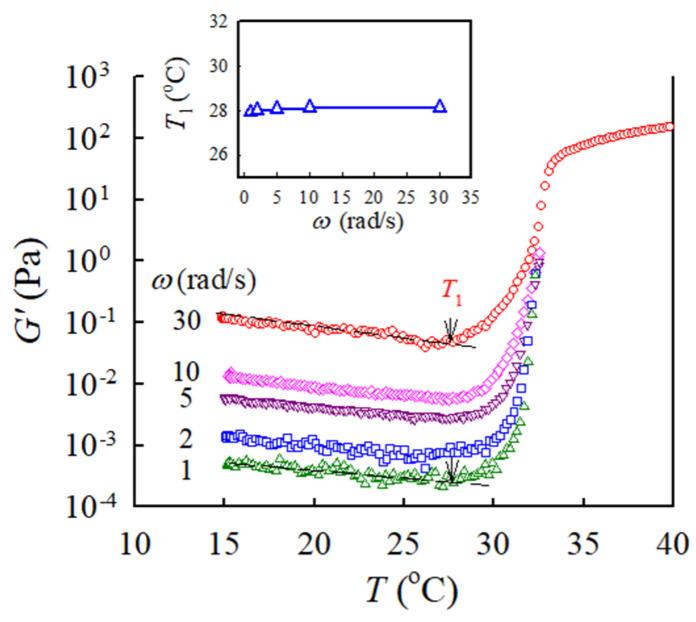
Effect of applied frequency on the temperature dependence of *G′* of 4 wt% a-PNIPAM solutions during temperature sweep at a heating rate of 0.2 °C/min. Arrows indicate the onset of *G′* upturn. Inset shows the frequency effect on the determined *T*_1_.

For the present a-PNIPAM/water solution, which is a dynamically asymmetric system [29,30], the shear-induced demixing will occur, provided that the applied frequency is sufficiently high to generate a large external strain rate to substantially exceed the intrinsic relaxation rate of polymer chains [30,31]. Figure 7 shows the frequency effect on the 4 wt% solution subjected to the same heating rate and strain amplitude. Slightly scattered data are observed for the low frequency of 1 and 2 rad/s because of the small torques. As expected, *G′* is larger when a higher frequency is applied. Regardless of the applied frequency (1–30 rad/s), the derived *T*_1_ remains constant at 28.0 °C, as shown in the inset. Similar trends were obtained for the 12 wt% solution. Thus, shear-induced demixing does not occur in the studied solutions of 4–12 wt% when subjected to a frequency of 5 rad/s.

### 2.4. Effect of Solution Concentration on the Rheological Properties

**Figure 8:**
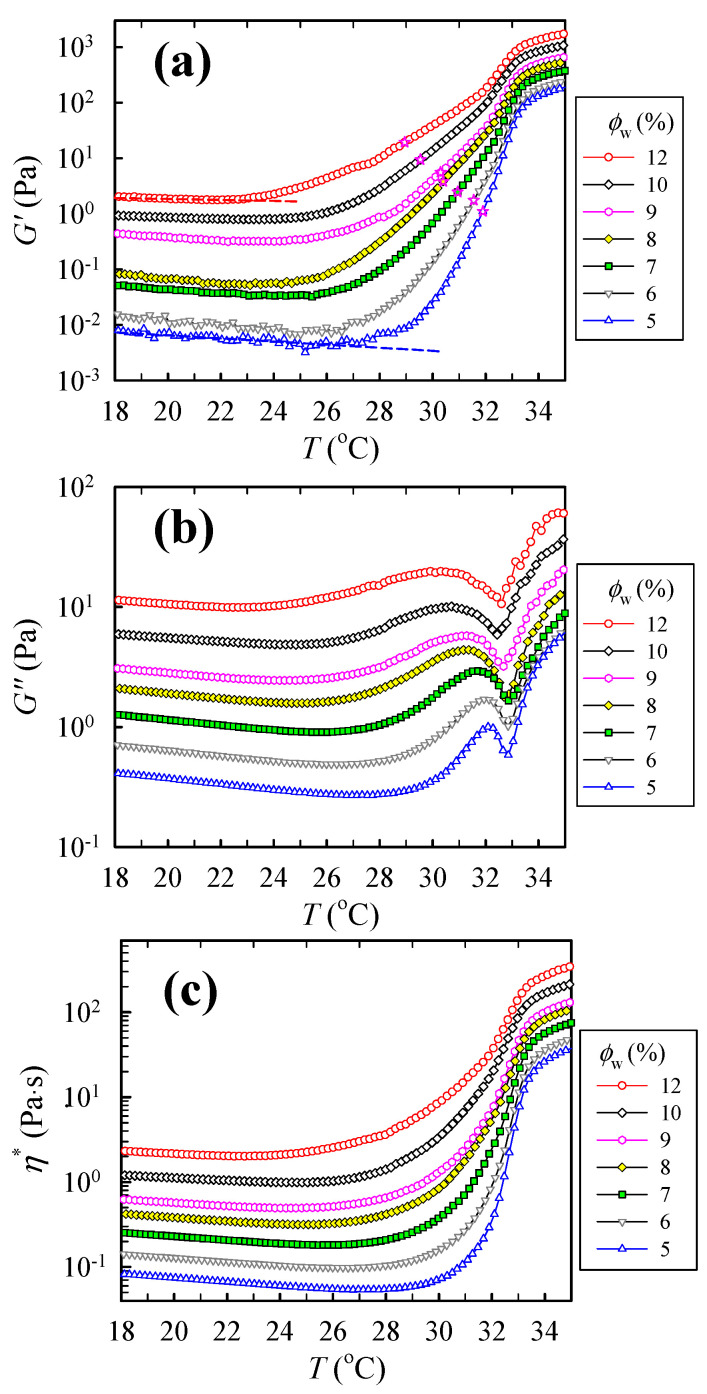
Temperature dependence of (**a**) storage modulus *G′*, (**b**) loss modulus *G″*, and (**c**) complex viscosity η* of a-PNIPAM aqueous solutions of different concentrations during temperature sweep at a heating rate of 0.2 °C/min and applied frequency of 5 rad/s. In (**a**), *T*_cross_ is represented by the ☆ symbol.

The heating rate of 0.2 °C/min and the applied frequency of 5 rad/s seem appropriate to extract the rheological properties of a-PNIPAM/water solutions under the temperature sweep experiment. Using these parameters, the measured *G′* and *G″* curves for the a-PNIPAM solutions with different concentrations are shown in Figure 8a,b, respectively. The *T*-dependence of the complex viscosity η*, calculated using the equation of (*G′*^2^ *+ G″*^2^*)*^0.5^*/ω*, is also displayed in Figure 8c for comparison. When the solution temperature approaches the spinodal point, the concentration fluctuations become significant, which is manifested in the elastic response to give rise to the *G′* increase with *T* at *T* > *T*_1_. As the solution concentration is increased, both the *T*_1_ and *T*_cross_ are decreased, and the *T*_min,G”_ is intact. The effect of concentration fluctuations is more pronounced on the measured *G′* than the other two quantities of *G″* and η*.

### 2.5. Determination of Spinodal Temperature Based on the Fredrickson–Larson–Ajji–Choplin Mean Field Theory

Fredrickson and Larson [32] developed a theoretical background based on the mean-field theory to disclose the contribution of the critical concentration fluctuations to the *G′* and *G″* in block copolymers during the order-disorder transition. They presented the contribution of the critical fluctuations to the linear viscoelastic of *G′*(*ω*,*T*) and *G″*(*ω*,*T*) based on the static structure factor *S*_0_(*q;T*) and the Onsager kinetics coefficient *λ*(*q;T*) for the two-component polymer systems. Their theoretical predictions validated the rheological measurements by Bates [33], who showed the anomalous viscoelastic behavior of block copolymer in the pre-transitional regime from the one-phase disorder to the two-phase order state. After introducing the random phase approximation for the static structure factor, Ajji and Choplin [20] applied the Fredrickson–Larson theory to the two-component polymer blends in the single-phase melt near critical point where the fluctuation contributions to *G′*(*ω*,*T*) and *G″*(*ω*,*T*) are anticipated to be dominant. *S*_0_(*q;T*) and *λ*(*q;T*) for the blends in the small *q*-range are given by

(1) $$S_{0}^{-1}\left(q;T\right)=2\left(\chi_{s}-\chi \right)+F_{1}q^{2}$$

(2) $$\lambda^{-1}\left(q;T\right)=F_{2}+F_{3}q^{2}$$

where

(3) $$F_{1}\equiv \frac{1}{\phi N_{1}}\frac{R_{g,1}^{2}}{3}+\frac{1}{\left(1-\phi \right)N_{2}}\frac{R_{g,2}^{2}}{3}$$

(4) $$F_{2}\equiv \frac{1}{\phi a_{1}^{2}W_{1}}+\frac{1}{\left(1-\phi \right)a_{2}^{2}W_{2}}$$

and

(5) $$F_{3}\equiv \frac{R_{g,1}^{2}}{\phi a_{1}W_{1}}+\frac{R_{g,2}^{2}}{\left(1-\phi \right)a_{2}W_{2}}$$

Here, *ϕ* is the volume fraction of the component 1; *R*_g,K_ and *N*_K_ (*K* = 1, 2) are the radius of gyration and the DP (degree of polymerization) of the *K*-th component, respectively; *a*_K_ and *W*_K_ (*K* = 1, 2) are the segmental length and the rate of segmental reorientation of the *K*-th component, respectively. *F*_1_ is a function of static parameters, whereas *F*_2_ and *F*_3_ depend on the dynamic parameters *W*_K_ which depend on *T*. Equations (1) to (5) lead to *G′*(*ω*,*T*) and *G″*(*ω*,*T*) given by

(6) $$G^{\prime }\left(\omega \right)=\frac{k_{B}T\omega^{2}}{1920\pi }F_{1}^{1/2}F_{2}^{2}{\left[2\left(\chi_{s}-\chi \right)\right]}^{-5/2}$$

(7) $$G^{\prime \prime }\left(\omega \right)=\frac{k_{B}T\omega }{240\pi }F_{1}^{-1/2}F_{2}{\left[2\left(\chi_{s}-\chi \right)\right]}^{-1/2}$$

where *k*_B_ is the Boltzmann constant. It is stressed that the quality *G″*(*ω*,*T*)^2^/*G′*(*ω*,*T*) depends only on *F*_1_, the statics of the concentration fluctuations but not on *F*_2_ and *F*_3_, the dynamics. Combined Equations (6) and (7) lead to

(8) $${\left[\frac{G^{\prime \prime }{\left(\omega \right)}^{2}}{G^{\prime }\left(\omega \right)T}\right]}^{2/3}={\left(\frac{k_{B}}{30\pi }\right)}^{2/3}F_{1}^{-1}\left[2\left(\chi_{s}-\chi \right)\right]$$

The temperature dependence of interaction parameter *χ* is generally expressed as *χ* = *A* + *B*/*T*, where *A* and *B* are constants. *B* is negative for the present system with LCST phase separation. By substituting *χ* in Equation (8), the following equation is obtained:

(9) $${\left[\frac{G^{\prime \prime }{\left(\omega \right)}^{2}}{G^{\prime }\left(\omega \right)T}\right]}^{2/3}=2\alpha BF_{1}^{-1}\left(\frac{1}{T_{s}}-\frac{1}{T}\right)$$

where *α* = (*k*_B_/30*π*)^2/3^ and *T*_s_ is the spinodal temperature. Based on Equation (9), the plot of (*G”*^2^/*G′T*)^2/3^ versus 1/*T* enables to access *T*_s_, provided that the concentration fluctuation contribution to *G′* and *G”* are dominant. In Equation (9), the term *F*_1_ is constant for a given polymer solution with no associating chains since *N*_K_ and *R*_g,K_ are fixed, as defined in Equation (3). Thus, a linear dependence of (*G”*^2^*/G’T*)^2/3^ versus 1/*T* is expected near the spinodal temperature in the single-phase solution, and *T*_s_ can be derived by extrapolating the linear dependence to (*G”*^2^*/G’T*)^2/3^ = 0 and finding the intercept value (1/*T*_s_) with 1/*T* axis without knowing the exact values of *B* and *F*_1_. Successful application of the Fredrickson–Larson–Ajji–Choplin (FLAC) theory to determine the *T*_s_ for polymer blends [20,22,23,24] and polymer solution [21] has been reported, and the derived *T*_s_ is in fair agreement with that obtained from the scattering approach.

However, for the present gelling solution, in which a-PNIPAM chains are able to associate with one another to develop the multimers for the gel network, the application of Equation (9) is not straightforward and requires verification. In the associating systems, unimers (single unassociated chains) as well as dimers, rimers, …, and, etc., of a-PNIPAM chains coexist; their mole fractions may change with *T* in such a way that the fraction of unimers tends to decrease but that of multimers to increase with increasing *T*. The existence of the multimers certainly affects the function of *F*_1_ to vary since the DP and chain configuration are changed as the solution temperature is raised. In this case, the structure factor for the associating chains is discussed in the Appendix in Ref. [14], and the function of *F*_1_ is derived to be

(10) $$F_{1}=\frac{1}{3}\frac{\langle R_{g,A}^{2}\rangle }{\langle mN\rangle \phi_{p}}$$

where $\langle R_{g,A}^{2}\rangle$ and 〈mN〉 are the average square of the radius of gyration and the average DP of the associated chains in the gelling solution at *T*. The definitions of 〈mN〉 and $\langle R_{g,A}^{2}\rangle$ are given in Equations (11) and (12), respectively [14].

(11) $$\langle mN=\rangle \frac{\sum_{1}^{m}\left(mN\right)\phi_{m}}{\sum_{1}^{m}\phi_{m}}$$

(12) $$\langle R_{g,A}^{2}\rangle =\frac{\sum_{1}^{m}\left(mN\right)\phi_{m}\langle R_{g,m}^{2}\rangle }{\sum_{1}^{m}\left(mN\right)\phi_{m}}$$

In Equations (10)–(12), *m* is the association number of the chains and *ϕ*_m_ and $\langle R_{g,m}^{2}\rangle$ are the concentration and mean square radius of gyration of the *m*-mer, respectively. The summation of *ϕ*_m_s with *m* gives the total polymer content in the solution, i.e., $\phi_{p}=\overset{m}{\underset{1}{\sum }}\phi_{m}$. 

The magnitude of *F*_1_ may not be constant because the number of associating chains changes with *T*, thereby giving rise to the *T* dependence of 〈mN〉 and $\langle R_{g,A}^{2}\rangle$. The aqueous solutions of a-PNIPAM develop the macroscopic gel network at *T*= *T*_gel_ below *T*_cp_, suggesting that the number of associating chains to form a *m*-mer increases with *T* to a large number of *m*_gel_ at *T*_gel_ and that a further increase of *m* with *T* at *T*_gel_ < *T* < *T*_cp_ may be small compared with *m*_gel_. Under this condition, *F*_1_ is effectively independent of *T* at *T*_gel_ < *T* < *T*_cp_. Accordingly, measured (*G”*^2^*/G’T*)^2/3^ as a function of *T* (>*T*_gel_) enables to assess *T*_s_, specifically from a perspective that the interchain associations strongly enhance the fluctuations, thereby giving rise to the extremely dominant contribution to *G’* and *G”*.

**Figure 9:**
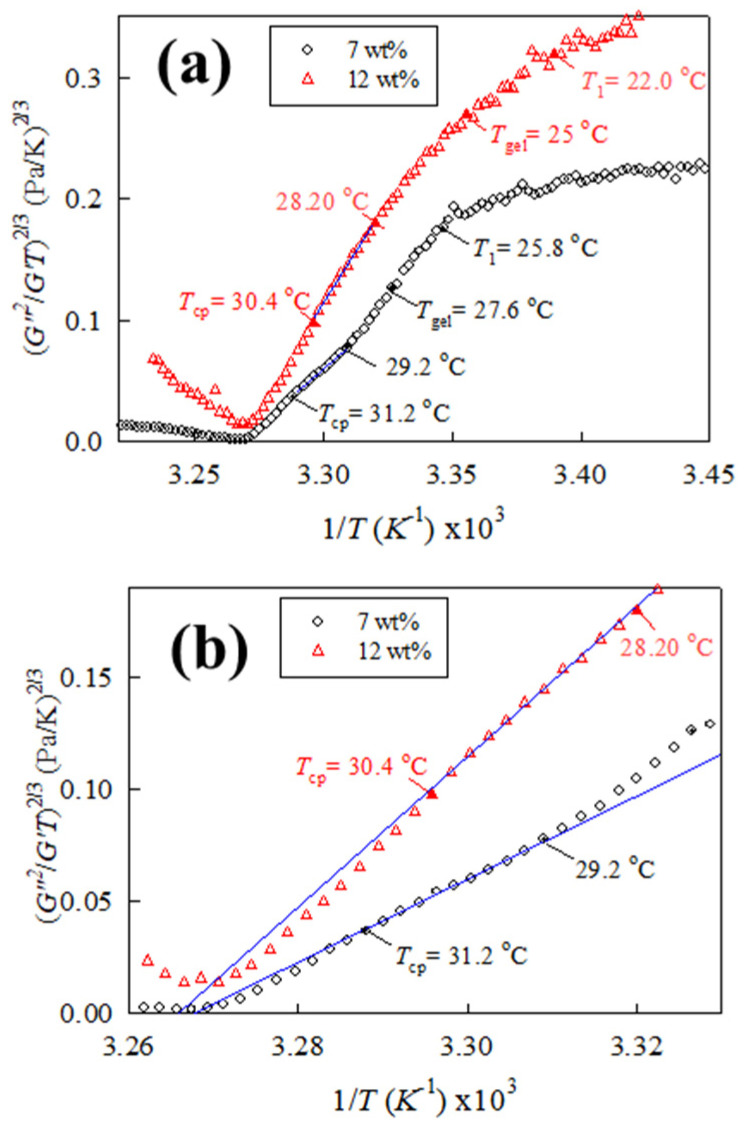
Determination of spinodal temperature of (**a**) 7 and 12 wt% a-PNIPAM solutions from linear dependence of (*G”^2^*/*G’T*)^2/3^ versus 1/*T*, and (**b**) enlarged portion of (**a**) for illustrating the linear regression to determine *T*_s,gel_, which can be evaluated by extrapolating the linear dependence to (*G”^2^*/*G’T*)^2/3^ = 0 in the temperature range *T*_gel_ < *T* < *T*_cp_ to obtain the intercept with 1/*T* axis, equaling to the reciprocal of the spinodal temperature, *T*_s,gel_. Linear regression is performed for temperatures above 29.2 and 28.2 °C for the 7 and 12 wt% solutions, respectively, giving rise to *T*_s,gel_ of 32.6 and 32.9 °C.

Based on Equation (9), the rheological properties of the 7 and 12 wt% solutions are further analyzed, and the corresponding plots are provided in Figure 9a. In addition, the respective values of *T*_1_, *T*_gel_, and *T*_cp_ are included to reveal the evolution of the (*G”*^2^*/G’T*)^2/3^ curves as the one-phase solution reaches the pre-gel regime (*T* > *T*_1_), transparent gel regime (*T*_gel_ < *T* < *T*_cp_), and opaque gel regime (*T* > *T*_cp_), respectively. For the 7 wt% solution, a linear regression line is feasibly obtained at a first glance at temperatures higher than 25.8 °C (=*T*_1_). However, a careful examination of the curve reveals that the linear slope at 1/*T*_cp_ < 1/*T* < 1/*T*_1_ is not constant. The deviation in the linear slope in different temperature ranges is because of the possible interchain associations, thereby leading to the fact that the parameter *F*_1_ is not constant but changes with temperature. The phenomenon is more evident for the curve of the 12 wt% solution. 

Hence, *T*_s,gel_ is the *T*_s_ evaluated by extrapolating the plot using the data at *T* > *T*_gel_ for the macroscopic gel to account for this deviation. The final linear slope at 1/*T*_cp_ < 1/*T* < 1/*T*_gel_ is extracted to derive a meaningful spinodal temperature of the a-PNIPAM gel, as shown by the blue solid line in Figure 9b; the extrapolated intercept gives rise to *T*_s,gel_ of 32.6 °C for the 7 wt% solution. For the 12 wt% solution, linear regression is deduced at temperatures above 28.2 °C (ca. 6 °C higher than the *T*_1_ of 22 °C) to derive the *T*_s,gel_ of 32.9 °C. Interestingly, the derived *T*_s,gel_ is rather close to the experimentally observed *T*_min,G”_ regardless of solution concentrations (Table 1), thereby implying a close correlation between the *T*_min,G”_ and the derived *T*_s,gel_. An abrupt increase in *G′* is seen at temperatures above *T*_min,G”_ (Figure 2 and Figure 3). The second uprise of *G′* and *G″* is attributed to the sudden formation of bicontinuous phase-separated domains associated with spinodal decomposition (SD). For the dynamically asymmetric solution of PNIPAN/water in a deep quench, the growth of the “3D percolated domain network” [34] composed of the gel network will further enhance the solution elasticity with raising *T*.

The correlation length *ξ* of polymer solutions associated with the concentration fluctuations is increased when the solution temperature gradually approaches the spinodal point. The temperature dependence of *ξ* can be expressed as [20,21]

(13) $$\xi^{2}=F_{1}{\left[2\left(\chi_{s}-\chi \right)\right]}^{-1}={\left(\frac{k_{B}}{30\pi }\right)}^{2/3}{\left[\frac{G^{\prime }\left(\omega \right)T}{G^{\prime \prime }{\left(\omega \right)}^{2}}\right]}^{2/3}$$

**Figure 10:**
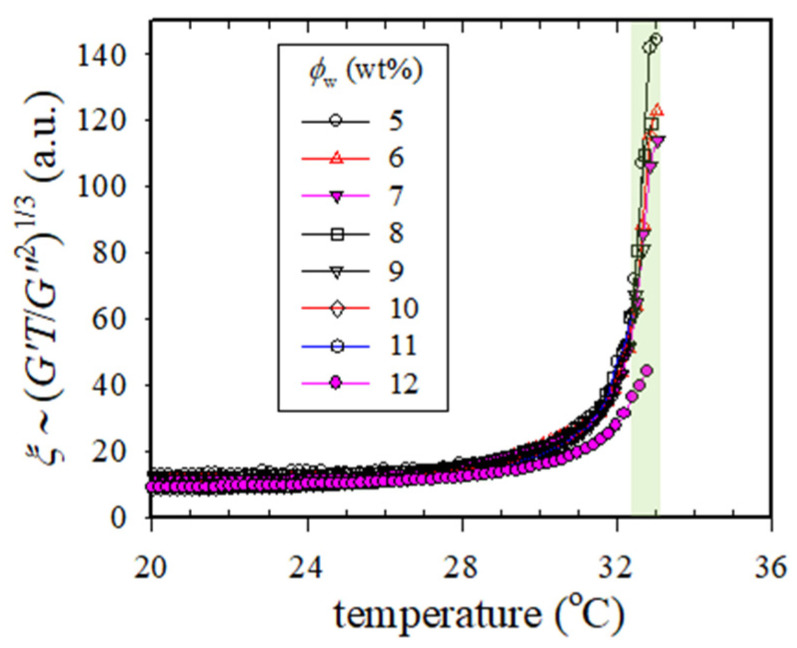
Plots of correlation length *ξ* versus *T* for the a-PNIPAM aqueous solutions with different concentrations. *ξ* theoretically diverges at *T*_s,gel_. The shaded area is the *T*_s.gel_ range for the a-PNIPAM solutions with a concentration range of 5–12 wt%.

For a given solution, the temperature dependence of *ξ* can be calculated from the corresponding *G’* and *G”*. Theoretically, *ξ* diverges at *T*_s_. Figure 10 shows the plots of (*G′T*/*G″*^2^)^1/3^ versus *T* for the solutions studied. The increase in *ξ* is observed at temperatures above 30 °C, and the value of *ξ* becomes maximum at *T*_min,G”_. These plots confirmed that regardless of solution concentrations (5–12 wt%), all the solutions exhibit a similar *T*_s,gel_ at 32–33 °C (shaded area in Figure 10).

We have performed a detailed analysis of SAXS of the 12 wt% a-PNIPAM aqueous solution to extract the *T*_s,gel_ after subtracting the excess scattering caused by the gel junctions from the observed scattering profile [14]. In this manner, the deduced *T*_s,gel_ by SAXS is 31.3 °C, which is higher than the *T*_s_ of a-PNIPAM solution before physical gelation (~29.3 °C). The *T*_s,gel_ derived from rheometry is higher than that obtained from the static scattering approach as compared to the results obtained from both approaches. Our determined values of *T*_s,gel_ are further compared with those reported in the literature without addressing the issue of physical gelation. *T*_s_ of PNIPAM aqueous solutions are determined from the Cahn linearized theorem to be 32.0–33.2 °C for the 4.4–7.3 wt% solutions based on the light scattering results [19]. Shibayama et al. [18] applied SANS to the 5.6–21.3 wt% PNIPAM/D_2_O solutions, yielding *T*_s_ values in the range of 33.1–35.1 °C. These results also show that *T*_s_ is relatively independent of the PNIPAM concentration. The LCST phase diagram of PNIPAM/H_2_O exhibits a bottom square-like shape, and this peculiar phase boundary has been theoretically validated by Okada and Tanaka [10] through the cooperative hydration hypothesis.

### 2.6. Phase Diagram of a-PNIPAM/water Based on Rheometry and Turbidity

For the so-called “off-critical” a-PNIPAM/H_2_O solutions of 5–12 wt% studied based on Ref. [3] (*ϕ*_c_ ~16 wt%), concentration fluctuations play a key role in the structure evolution when *T*_s_ is approached below *T*_b_. The thermally-induced concentration fluctuations may occur over a wide range of length scales from ~nm to ~μm. The magnitude and length scale of concentration fluctuations both diverge at *T*_s_. Thus, we propose the following phase transitions on heating the a-PNIPAM/H_2_O solution based on our rheological results.

At temperatures higher than *T*_1_, the concentration fluctuations in the single-phase solution become effective to yield interchain associations. However, the small length-scale fluctuation has only a small amplitude and rapidly decays. It barely facilitates intersegmental contacts and interchain associations. This is because a small-scaled region (~nm) rich in chain segments is stochastically hardly probable and thereby insignificant from the viewpoint of statistical mechanics. On the contrary, the large length-scale fluctuation has a very large amplitude of fluctuation and very slowly decays. It creates local regions where the local concentration of polymers is much higher than the average concentration. Moreover, these regions survive for a long time. These physical factors stochastically greatly facilitate the diffusion-controlled interchain associations mediated by attractive interactions between the hydrophobic groups to form the multimers. The rate of hydrophobic association is higher than the rate of hydrophobic dissociation, thereby giving rise to the formation of the associations.

The association mediated by the concentration fluctuations is diffusion-controlled in the regions rich in polymers where the local concentration is conceivably much higher than *ϕ*_e_. The small multimers (e.g., dimers and trimers) built up by intermolecularly associated single chains act as gel junctions and primary building blocks of the network strands which eventually build up the macroscopic physical gel network at *T*_gel_. The transparent gel turns opaque as the temperature is raised to *T*_b_. When the temperature is further raised to *T*_s,gel_ or equivalently to *T*_min,G”_, the opaque solution confined in the soft gel undergoes a large-scale (~μm) viscoelastic phase separation.

**Figure 11:**
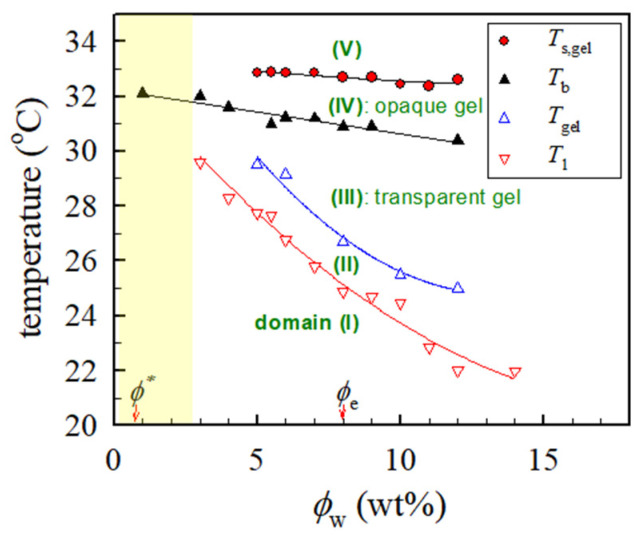
Phase diagram of a-PNIPAM/H_2_O binary mixtures. *T*_1_ and *T*_s,gel_ are obtained in this work from the temperature sweep test at a heating rate of 0.2 °C/min and applied frequency of 5 rad/s. *T*_gel_ and *T*_b_ are taken from Refs. [14,15]; *T*_gel_ derived from the Winter-Chambon criterion and *T*_b_ derived from the extrapolated *T*_cp_ at a zero-heating rate. The overlap concentration ϕ* is ~0.6 wt% and the entanglement concentration *ϕ*_e_ is ~8 wt%.

Figure 11 shows the phase diagram of a-PNIPAM aqueous solutions assessed by the linear viscoelasticity and turbidity. The corresponding values of *T*_1_, *T*_gel_, *T*_b_, and *T*_s,gel_ are included. However, the rheological properties of solutions at concentrations <3 wt% were not measured because of apparatus limitations; thus, the related data are absent, as represented by the shaded area. Five solution domains are identified in the quasi-equilibrium phase diagram depending upon the composition and temperature, namely, domains (I): *T* < *T*_1_, stable single-phase solution with the thermorheological simplicity, (II): *T*_1_ < *T* < *T*_gel_, the single-phase solution containing the interchain association subjected to profound concentration fluctuations, (III): *T*_gel_ < *T* < *T*_b_, transparent gel, (IV): *T*_b_ < *T* < *T*_s,gel_, opaque gel, and (V): *T* > *T*_s,gel_, unstable gel undergoing viscoelastic phase separation via SD. The demarcation was primarily based on the dynamic temperature sweep test. A more advanced work of static scattering experiments is required to clarify the details of the structural evolution of the developing a-PNIPAM gels to extract the *T*_s,gel_ to construct the “thermodynamic” phase diagram.

## 3. Conclusions

Herein, detailed rheological measurements on the semidilute solutions of a-PNIPAM/H_2_0 with a wide concentration of 5–12 wt% were performed to cover the unentangled and entangled solution regimes. For the binary mixtures, physical gelation was validated to occur at a temperature (*T*_gel_) lower than the binodal temperature (*T*_b_). Analyses of the temperature dependence of rheological data of *G’* and *G”* obtained from the dynamic temperature sweep test gave rise to two important transition temperatures, such as the pre-gel temperature (*T*_1_) and spinodal temperature of a-PNIPAM hydrogel (*T*_s,gel_). *T*_1_ was directly deduced from the *T*-dependence of the *G’* curve, at which the first upturn of *G’* was detected upon heating, whereas *T*_s,gel_ was carefully derived based on the Fredrickson-Larson-Ajji-Choplin mean field theory. Based on these transition temperatures (*T*_1_, *T*_gel_, *T*_b_, and *T*_s,gel_), the phase diagram of a-PNIPAM/H_2_0 was successfully constructed to show the relative position of the LCST line and the sol-gel transition curve. Finally, our results showed that five phase domains of a-PNIPAM/H_2_O can be identified for the semi-dilute solutions on the temperature-composition diagram.

## 4. Materials and Methods

a-PNIPAM pellets were obtained from Scientific Polymer Products Inc. (USA) with the weight-average molecular weight and polydispersity of 6.58 × 10^5^ g/mol and 1.49, respectively, which were determined from gel permeation chromatography in DMF at 25 °C. De-ionized water was used as a solvent to prepare the solutions. First, a-PNIPAM pellets were vacuum-dried before solution preparation. Then, different amounts of polymers and solvents were vigorously mixed at 10 °C for 3 days to prepare the single-phase solutions of different concentrations, followed by storage in a freezer at 5 °C before measurements.

The linear viscoelastic properties of a-PNIPAM solutions were measured with a rheometer (ARES) using a cup-and-bob fixture under a small amplitude oscillatory shear mode. For semi-dilute solutions, the torque for the rheological measurement is low, particularly for the unentangled polymer solution. A fixture with a cup diameter, bob diameter, and bob length of 34, 32, and 34 mm, respectively, was used to obtain reliable torque for correct measurements. Thus, a sample volume of ~15 mL was necessary for measurements. A temperature sweep test at a given heating rate was performed to obtain the temperature dependence of the dynamic storage modulus *G′(ω)* and loss modulus *G″(ω)* to reveal the phase transition. The temperature varied from 10–40 °C, thus covering the entire range of solution behavior from homogeneous to phase-separated. The effects of heating rate and applied frequency (*ω*) on the measured curves were studied by changing the rate (0.2, 0.5, 0.8, 1.0, 1.5, and 2.0 °C/min) and frequency (1, 2, 5, 10, and 30 rad/s). Unless otherwise specified, the heating rate and applied frequency used in the temperature sweep test were 0.2 °C/min, and 5 rad/s, respectively. During the temperature sweep test, the appropriate strain amplitude was selected based on the preliminary strain sweep test to ensure the conditions of linear viscoelasticity and sufficient torque for precise data. The intrinsic viscosity [*η*] of the prepared solutions was measured at 20 °C using a capillary rheometer.

## Figures and Tables

**Table 1:** Transition temperatures of a-PNIPAM aqueous solutions with different concentrations derived from the rheological measurements.

*ϕ*_w_ (wt%)	*T*_1_ (°C)	*T*_gel_ (°C)	*T*_s,gel_ (°C)	*T*_min,G”_ (°C)
5	27.7	29.5	32.8	32.9
6	26.8	29.1	32.8	32.9
7	25.8	27.6	32.6	32.9
8	24.9	26.7	32.6	32.7
9	24.7	26.5	32.7	32.7
10	24.4	25.5	32.5	32.4
12	22.0	25.0	32.9	32.6

Note: *T*_1_, *T*_min,G”_, and *T*_s,gel_ are obtained in this work from the temperature sweep test at a heating rate of 0.2 °C/min and applied frequency of 5 rad/s, whereas *T*_gel_ is obtained from Refs. [14,15] by the isothermal frequency sweep test.

## Data Availability

Not applicable.
